# Physiological comparison of noninvasive ventilation and CPAP on inspiratory efforts after extubation in critically ill patients with morbid obesity: a post-hoc analysis

**DOI:** 10.1186/s13613-025-01603-3

**Published:** 2025-11-17

**Authors:** Martin Mahul, Mathieu Capdevila, Fabrice Galia, Audrey De Jong, Samir Jaber

**Affiliations:** 1 Department of Anesthesia and Intensive Care Unit, Regional University Hospital of Montpellier, St-Eloi Hospital, Montpellier, France; 2 Intensive Care Unit, Anesthesia and Critical Care Department, Nimes University Hospital, Nimes, France; 3 University of Montpellier, PhyMedExp, INSERM U1046, CNRS UMR, 9214, CEDEX 5 Montpellier, France

**Keywords:** Noninvasive ventilation, Obesity, Weaning, Extubation, Work of breathing

## Abstract

**Background:**

No study has evaluated the inspiratory effort in patients with obesity immediately after extubation according to the noninvasive ventilatory support used. We aimed to determine, in critically ill patients with morbid obesity, whether Non Invasive Ventilation applied with facial mask with Pressure Support above Positive End-Expiratory Pressure (PSV-PEEP) may reduce patient inspiratory efforts to a greater extent than Continuous Positive Airway Pressure (CPAP) after extubation.

**Methods:**

We conducted a post-hoc analysis based on data from a physiological study involving consecutive patients with morbid obesity prior to extubation. Flow, airway, esophageal, and gastric pressure signals were then recorded 20 min after extubation under three distinct conditions: (1) standard oxygen, (2) CPAP and (3) PSV-PEEP. Inspiratory efforts were assessed by calculation of the trans-diaphragmatic pressure (Pdi) and work-of-breathing (WOB).

**Results:**

Fifteen patients with mean body mass index of 45 kg/m^2^ (± 8 kg/m^2^) were enrolled. WOB and Swing Pdi were lower with PSV-PEEP than with CPAP and standard oxygen respectively 5.3 [3.6–6.0] vs 8.4 [7.4–10.0] and 14.9 [11.1–22.1] J/min (p < 0.001), and 5.9 [4.0–7.8] vs 11.4 [10.1–13.1] and 19.6 [18.5–23.6] cmH2O (p < 0.001). We also observed a significant decrease of respiratory rate (RR) and RR/V_T_ (tidal volume) ratio with the use of PSV-PEEP (24.4 [21.9–27.7] breaths/min and 65.7 [45.1–78.5] min/mL, respectively), and with the use of CPAP (24.6 [24.1–34.5] breaths/min and 75.3 [57.2–108.0] min/mL), compared with standard oxygen (29.0 [24.2–34.9] breaths/min and 81.1 [73.5–108.9] min/mL), p < 0.05.

**Conclusion:**

In critically ill post extubation patients with morbid obesity, both PSV-PEEP and CPAP reduced the inspiratory effort indexes including inspiratory work-of-breathing, traducing an unload of inspiratory muscles. This effect was more important when PSV-PEEP was used in comparison to CPAP, suggesting a more pronounced effect of inspiratory muscle unloading.

**Supplementary Information:**

The online version contains supplementary material available at 10.1186/s13613-025-01603-3.

## Introduction

Post-extubation period is a critical time during the intensive Care Unit (ICU) stay with up to 15–20% of extubation failure in high-risk population with an association of higher morbidity [1]. Post-extubation respiratory management of critically ill patients with obesity often represents a clinical challenge [2] because this population present specificities in overall [3, 4] and in ventilatory management [5]. Obesity decreases respiratory system compliance, lung volumes and functional residual capacity and may be associated with impaired upper airway function and neuromuscular strength [6]. Moreover, oxygen uptake of patients with obesity at rest, and even more during exercise, is widely increased, resulting in a higher level of work of breathing (WOB) [7, 8]. This excessive WOB, especially during weaning and post-extubation period where WOB is already often increased, may be a potential reason for extubation failure of critically ill patients with obesity [9, 10].

Noninvasive ventilation (NIV) has been considered as a reliable therapy by an International Consensus Conference to prevent respiratory failure after weaning [1, 11, 12] and has been proposed to prevent post-extubation failure in medical [13, 14] or surgical [15] ICU population. Moreover, recent studies [16–18] found a beneficial effect of administering prophylactic NIV immediately during post-extubation period in patients with obesity, with less incidence of acute respiratory failure and reintubation. NIV after early extubation also appeared associated with a reduction in total days spent on invasive mechanical ventilation [19]. NIV consists of the application of a positive pressure ventilation, usually with Pressure Support Ventilation above Positive End Expiratory Pressure (PSV PEEP) or with Continuous Positive Airway Pressure (CPAP). Both settings are well validated in obesity hypoventilation syndrome [20]. Physiologically, applying positive end-expiratory pressure (PEEP) to the airway opening has been shown to improve functional residual capacity and gas exchanges, especially in healthy population with obesity and obstructive sleep apnea [21]. While PSV-PEEP has demonstrated clinical and physiological benefits after extubation, this has not been shown with CPAP, which may nonetheless represent a better-tolerated alternative (no synchronization issues, improved comfort, easier implementation).

The aim of this physiological study was to compare the inspiratory effort between PSV-PEEP and CPAP, using standard oxygen as a baseline in critically ill patients with morbid obesity after extubation. We hypothesized that inspiratory muscle effort would be reduced using PSV-PEEP in comparison with CPAP and spontaneous breathing with standard oxygen.

## Methods

### Study

A post hoc analysis derived from a prospective, crossover, physiological study was performed. The original study was approved by the Ethics Committee of the Saint-Eloi Teaching Hospital (reference 2012 A-00294–39, Comité de Protection des Personnes Sud Méditerranée III, Montpellier, France) and registered on clinical trial.gov (reference NCT01616901). All patients provided their written informed consent. The authors designed the trial, collected the data, and performed the analyses. There was no industry support or involvement in the trial. All the authors revised the manuscript, vouched for the accuracy and completeness of the data, and approved the decision to submit the manuscript for publication.

### Patients

All consecutive patients with morbid obesity, defined by a body mass index over 35 kg/m^2^ [22], were considered eligible for inclusion in the study if they were older than 18 years of age, admitted to the ICU, covered by public health insurance, mechanically ventilated for at least 24 h, and were considered for extubation by the physician in charge, not involved in this research. Patients were not included if there was any contraindication for insertion of an esophageal catheter, anatomical factors precluding the use of NIV, or previous extubation during the same ICU stay with previous inclusion in the study.

### Experimental procedure and study design

Included patients received 5 different spontaneous trials in a random order before extubation, as described in the original study [10]. The post-extubation period was divided into three consecutive phases. First, as described in the original study [10], patients received standard oxygen through an oro-nasal facemask during 20 min, at 5 L/min corresponding to a fraction of inspired oxygen (FiO₂) of 0.4 [23]. They were then transitioned to CPAP at 7 cmH₂O for 20 min. This CPAP phase also allowed to optimize patient comfort and tolerance, and was part of our local protocol of introduction of NIV following extubation [24]. Finally, patients were switched to NIV PSV-PEEP with the same ventilatory settings as those prescribed prior to extubation as protocolized in our unit. Measurements of respiratory effort were recorded continuously, and for analysis the last 10 interpretable cycles were retained after 20 min of each phase.

### Measurements

All patients were studied in a semi-recumbent position with the head of the bed elevated to an angle from 30 to 45 degrees, to lessen the effort to breathe, according to patient's comfort [25]. The ventilator for all patients was a General Electric Engström® (Boston, MA, USA).

Airflow was measured with a pneumotachograph (Fleish no. 2; Fleisch, Lausanne, Switzerland) connected to a differential pressure transducer (MP45, ± 2 cmH2O; Validyne, Northridge, CA, USA) as previously described [10]. Minute ventilation (VE), tidal volume (V_T_), inspiratory time (Ti), expiratory time (Te), total cycle duration (Ttot), respiratory rate (RR), were calculated from the numerical integration of the flow signal. Because of leaks, expired tidal volume (V_Te_) was considered closer to the true V_T_ taken by the patient and was used for data analysis of inspiratory effort.

Pressure at the airway opening (Pao) was assessed via a side port connected to a pressure transducer. Esophageal (Pes) and gastric (Pga) pressures were measured using a double balloon-tipped catheter (Nutrivent, Sidam s.r.l.,Mirandola, Italy) positioned in the mid-esophagus and in the stomach and connected to two differential pressure transducers. Transdiaphragmatic (Pdi) pressure was obtained by subtracting Pes from Pga.

As previously described [26, 27], the patient’s inspiratory work of breathing (WOB) was calculated from Pes–VT loops using the Campbell diagram. The static chest-wall compliance was assumed at 4% of predicted vital capacity per cmH₂O. Particular attention was given to minimizing mask leaks, but as these may cause overestimation of inspired volume, inspiratory WOB was corrected by the ratio of expired to inspired minute volume, which was applied as a factor to the flow signal. Proper positioning of esophageal balloon (lower third portion of esophagus) was verified by an occlusion test previously described by Baydur et al. [28] while proper positioning of gastric balloon was verified by application of repeated manual gastric pressure on the patient's abdomen by the physician in charge to observe fluctuations in gastric pressure. Esophageal and diaphragmatic pressure–time products (PTPes and PTPdi) were also measured as previously reported [10, 29, 30]. Briefly, the PTP (esophageal or transdiaphragmatic) was obtained by measuring the area under the Pes or Pdi signal between the onset (beginning of the positive flow) and the end of inspiration and was referenced to the chest wall static recoil pressure–time relationship. Pes values at zero-flow were considered as beginning and end of inspiration and we considered that any difference between initial Pes and the zero-flow point indicates intrinsic PEEP [31]. The intrinsic PEEP was then corrected for expiratory muscle activity, as detected on Pga tracings [32].

### Outcomes

The primary outcome was the inspiratory effort assessed by WOB. Secondary outcomes were other indicators of inspiratory effort: swing Pdi, swing Pes, PTPdi and PTPes. Exploratory outcomes were RR, RR/V_T_, Te, PEEPi, leaks.

### Statistical analysis

All continuous variables are presented as median [interquartile range] and categorical variables as number (percentage). Based on expected reductions in inspiratory WOB across the three modalities of assistance [10] and assuming a large within-subject effect size (f ≈ 0.5), 12 patients were required to achieve 80% power at α = 0.05. Owing to the small number of patients (< 30), we used non-parametric analysis of variance and two-by-two tests. To assess differences between the 3 conditions, we used the Friedman test and then pairwise comparisons with Wilcoxon test if a significant difference appeared. The p-values were adjusted for multiple testing using the Holm method (equivalent to Dunn’s correction). Statistical analysis was performed by an independent statistician using R software © (R Foundation for Statistical Computing, Auckland, New Zealand).

## Results

### Patients characteristics

During the period of the study, from March 2012 to December 2012, 17 patients were included in the original study, of whom 15 were analyzed in this post-hoc study. One patient was excluded from the original cohort due to accidental removal of esophageal catheter before the end of recording, and one further patient was excluded from the post-hoc analysis due to accidental removal of esophageal catheter before the end of recording after extubation (Figure S1). There was 12 women and 3 men, the mean body mass index was 45 kg/m^2^ (± 8 kg/m^2^). Characteristics of the subjects are detailed in Table 1 and Table S1. Mean duration of invasive mechanical ventilation through endotracheal tube before enrollment in the study was 6 days (± 7 days). One patient presented extubation failure, with need for re-intubation in the 48 h after extubation. In accordance with our clinical practice for respiratory weaning in patients with morbid obesity, all patients included in the study received intermittent prophylactic NIV during the first 48 h after extubation.

**Table 1:** Characteristics of the patients

Characteristics	All patients (n = 15)
Female	12 (80)
Age, years	54 [49–68]
SAPS II	60 [46–67]
Height, cm	162 [155–168]
Weight, kg	115 [94–130]
BMI, kg/m^2^	43 [39–47]
Comorbidities	
Diabetes	4 (27)
Systemic hypertension	3 (20)
Cardiac insufficiency	1 (7)
Asthma	2 (13)
COPD	1 (7)
Obstructive sleep apnea	4 (27)
Etiology of respiratory failure	
Pneumonia	2 (13)
Severe acute abdominal disease	9 (60)
Septic shock	3 (20)
Severe asthma exacerbation	1 (7)
Endotracheal tube internal diameter	
7.5 mm	13 (87)
8 mm	2 (13)
Length of mechanical ventilation, days	4 [2–6]
PSV level before extubation, cmH_2_O	10 [8–12]
PEEP level before extubation, cmH_2_O	8 [7–8]
Extubation success	14 (93)
Survival	14 (93)

Continuous variables are presented as median [interquartile range] and categorical variables as number (percentage)

BMI: Body Mass Index; COPD: chronic Obstructive Pulmonary Disease; PEEP: Positive End Expiratory Pressure; PSV: Pressure Support Ventilation; SAPS II: Simplified Acute Physiology Score II (2)

### Inspiratory effort

**Fig. 1:**
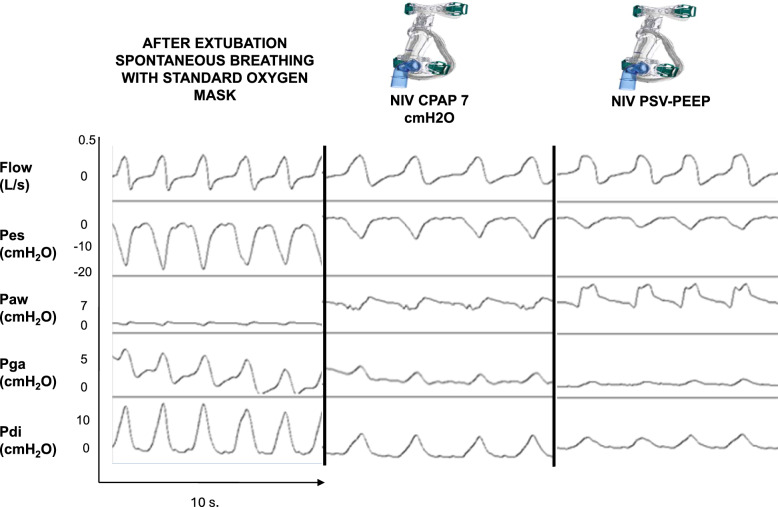
Ventilatory pattern during the three conditions. One patient is presented with the acquisition of flow (L/s), esophageal (Pes, cmH_2_O), airway (Paw, cmH_2_O), gastric (Pga, cmH_2_O) and diaphragmatic pressure signals (Pdi, cmH_2_O)

**Fig. 2:**
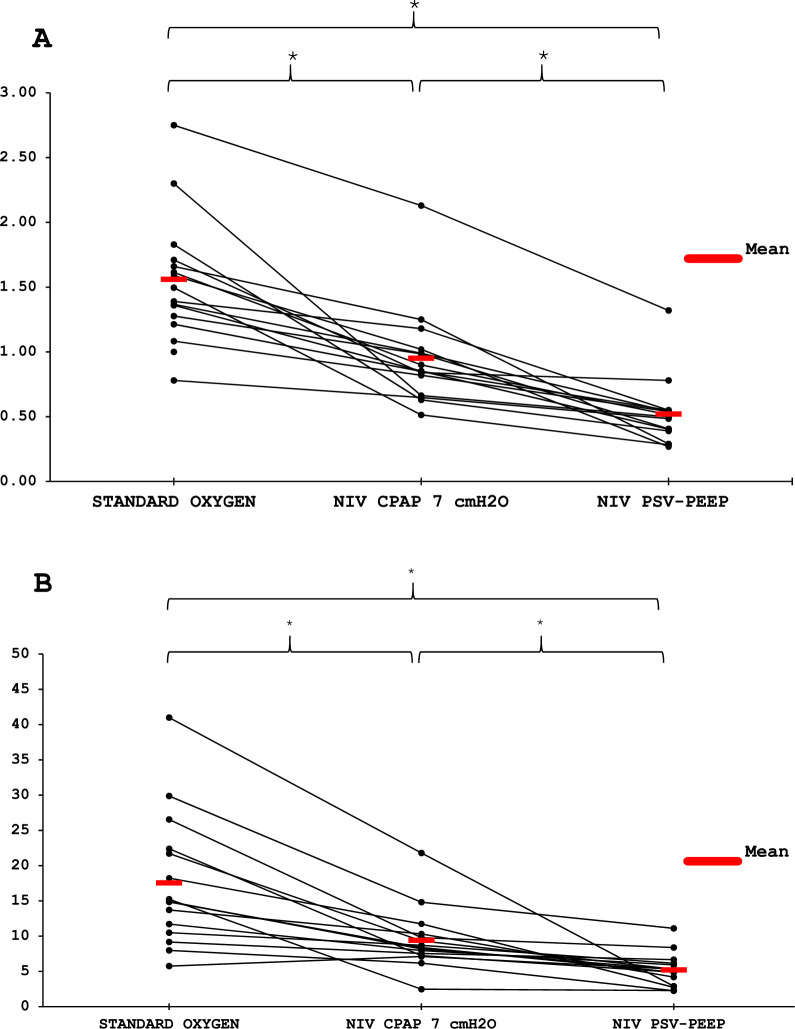
Individual and mean changes in Work Of Breathing in Joules/Liter (**A**) and in Joules/minute (**B**) during the three conditions. * p < 0.001

**Fig. 3:**
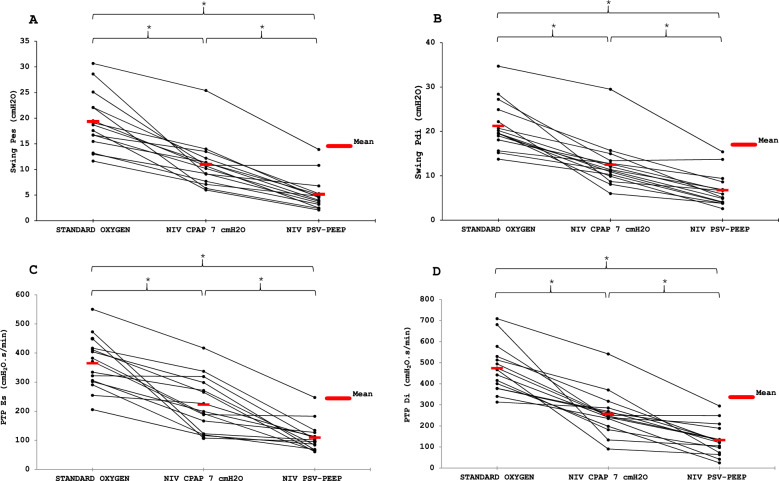
Individual and mean changes in esophageal (**A**) and diaphragmatic (**B**) swings, esophageal (**C**) and diaphragmatic (**D**) Pressure Time Products during the three conditions. * p < 0.001

The inspiratory effort required after extubation was lower with PSV-PEEP than with CPAP, and for both the effort was lower than spontaneous breathing with standard oxygen. Respectively the WOB 5.3 [3.6–6.0], 8.4 [7.4–10.0] and 14.9 [11.1–22.1] J/min (p < 0.001) and Swing Pdi was 5.9 [4.0–7.8], 11.4 [10.1–13.1] and 19.6 [18.5–23.6] cmH2O (p < 0.001) with PSV-PEEP, CPAP and standard oxygen (Table 2A). Figure 1 presents the ventilatory pattern during the three studied conditions in an illustrative patient showing the decrease in respiratory effort (Pes and Pdi) with respiratory support (PSV-PEEP and CPAP). Figures 2, 3 show the individual and mean values of the main inspiratory effort parameters studied. As presented in Table 2A, Table S2 and Table S3, for all inspiratory effort parameters (WOB, esophageal and diaphragmatic swings, esophageal and diaphragmatic PTP) there was a significant difference between PSV-PEEP and spontaneous breathing with standard oxygen, between CPAP and spontaneous breathing with standard oxygen, and between PSV-PEEP and CPAP (p < 0.001). The observed decrease in inspiratory effort was more important when PSV-PEEP was used in comparison to CPAP.

**Table 2:** Respiratory muscle work during the three conditions

	After Extubation Spontaneous Breathing	NIV-CPAP 7 cmH_2_O	NIV PSV + PEEP
WOB, J/L	1.5 [1.3–1.7]	0.8 [0.7–1.0]*	0.5 [0.4–0.5]*#
WOB, J/min	14.9 [11.1–22.1]	8.4 [7.4–10.0]*	5.3 [3.6–6.0]*#
Swing Pes, cmH_2_O	18.7 [16.1–22.1]	10.7 [8.4–11.8]*	4.5 [3.3–5.2]*#
Swing Pdi, cmH_2_O	19.6 [18.5–23.6]	11.4 [10.1–13.1]*	5.9 [4.0–7.8]*#
PTP es, cmH_2_O.s/min	371 [303–414]	199 [145–285]*	101 [76–120]*#
PTP di, cmH_2_O.s/min	467 [389–521]	247 [216–277]*	126 [85–162]*#

Continuous variables are presented as median [interquartile range]

CPAP: continuous positive airway pressure; NIV: non-invasive ventilation; Pdi: trans-diaphragmatic pressure; PEEP: Positive End Expiratory Pressure; Pes: esophageal pressure; PSV: pressure support ventilation; PTPdi: trans-diaphragmatic pressure time product; PTPes: esophageal pressure time product; PSV: pressure support ventilation; WOB: work of breathing

^*^p < 0.001 when compared with spontaneous breathing (Wilcoxon-Holm)

^#^p < 0.001when compared with CPAP 7 cmH2O (Wilcoxon-Holm)

### Respiratory parameters

Figures S2 and Figure S3 show the individual and mean values of RR and RR/VT. Table 3 shows a significant decrease of RR and RR/V_T_ ratio with the use of PSV-PEEP (24.4 [21.9–27.7] breaths/min and 65.7 [45.1–78.5] min/mL, respectively) when compared with spontaneous breathing and CPAP and with the use of CPAP (24.6 [24.1–34.5] breaths/min and 75.3 [57.2–108.0] min/mL) when compared with spontaneous breathing (29.0 [24.2–34.9] breaths/min and 81.1 [73.5–108.9] min/mL) p < 0.05. V_T_ and VE remaining identical for all the 3 conditions. Applying a positive pressure (PSV-PEEP or CPAP) was associated with a significant reduction of PEEPi and an increase of Te when comparing to spontaneous breathing. There was no significant difference in PEEPi or Te results between PSV-PEEP or CPAP. Using PSV-PEEP was not significantly associated with an increase in leaks when compared with CPAP.

**Table 3:** Respiratory Parameters during the three different conditions

	After Extubation	NIV-CPAP 7 cmH_2_O	NIV PSV + PEEP
V_Te_, L	0.32 [0.30–0.40]	0.37 [0.28–0.48]	0.39 [0.34–0.50]
VE, L/min	10.2 [8.5–11.3]	8.9 [8.6–11.5]	10.6 [9.2–11.1]
Leaks, %	NA	32.0 [20.0–45.0]	28.0 [18.0–39.0]
RR, breaths/min	29.0 [24.2–34.9]	24.6 [24.1–34.5] *	24.4 [21.9–27.7]*#
RR/V_T_, min/mL	81.1 [73.5–108.9]	75.3 [57.2–108.0]	65.7 [45.1–78.5]*#
PEEPi, cmH₂O	2.0 [0.9–3.0]	0.90 [0.65–1.40]*	0.50 [0.35–1.20]*
Ti, s	0.70 [0.60–1.05]	0.90 [0.80–0.95]	0.90 [0.80–1.00]
Te, s	1.20 [1.00–1.40]	1.40 [1.05–1.60]*	1.50 [1.35–1.85]*
Ttot, s	2.00 [1.70–2.50]	2.40 [1.75–2.50]	2.50 [2.20–2.70]*
Ti/Ttot, %	41.2 [35.9–43.8]	38.8 [35.3–43.8]	36.5 [34.7–38.7]*

Continuous variables are presented as median [interquartile range]

CPAP: continuous positive airway pressure; NIV: non-invasive ventilation; PSV: pressure support ventilation; PEEP: Positive End Expiratory Pressure; PEEPi: intrinsic positive end-expiratory pressure; RR: respiratory rate; Ti: inspiratory time; Te: expiratory time; Ttot: total respiratory time; VE: volume per minute; V_Te_: expired tidal volume

^*^p < 0.001 (< 0.05 for RR and RR/VT) when compared with spontaneous breathing (Wilcoxon-Holm)

^#^p < 0.001 (< 0.05 for RR and RR/VT) when compared with CPAP 7 cmH2O (Wilcoxon-Holm)

## Discussion

The main results of this study can be summarized as follows: in patients with morbid obesity, when compared to spontaneous ventilation with face mask, NIV with PSV-PEEP and CPAP settings reduced the inspiratory WOB, traducing an unload of inspiratory muscles; with a more pronounced effect on WOB when using PSV-PEEP than CPAP. These findings were consistent with all inspiratory effort indexes. To our knowledge, this is the first physiologic study to assess the effects of CPAP and PSV-PEEP on respiratory mechanics and inspiratory efforts of patients with morbid obesity during immediate post-extubation period.

Earlier investigations [33] suggested that the prophylactic use of NIV in patients with morbid obesity during the first 24 h post-operatively significantly decrease pulmonary dysfunction after gastroplasty and accelerated recovery to pre-operative pulmonary function. This improvement was attributed to a combined effect of improved lung inflation, prevention of alveolar collapse and reduced inspiratory threshold load. De Jong et al. [16] also highlighted that for patients with obesity, early post-extubation prophylactic NIV reduced the frequency of respiratory failure. This multicenter clinical trial [16] involving critically ill adults with obesity undergoing endotracheal extubation compared the effectiveness of NIV with various oxygen therapy methods, including high-flow nasal oxygen (HFNO) and standard oxygen, in terms of reducing treatment failure. The use of NIV was associated with significantly lower treatment failure rates compared to the use of oxygen therapy, regardless of whether the oxygen therapy was delivered through HFNO or standard oxygen. Moreover, the positive effects of NIV were consistent across different subgroups of patients. These subgroups were defined by factors such as the presence of SARS-CoV-2 infection, type of admission, and the length of mechanical ventilation. A post hoc analysis was performed to assess the impact of reallocating patients who were initially receiving oxygen therapy but were switched to NIV as rescue therapy. This analysis revealed a significantly lower rate of reintubation within 3 days among patients who were in the NIV group after this reallocation. These findings [16] emphasize the potential benefits of NIV in this specific patient population. These results are consistent with the post hoc analysis by Thille et al. [17], which demonstrated beneficial effects of NIV in obese or overweight patients included in a randomized trial originally designed for high-risk populations [13]. Classifying patients with obesity as inherently high-risk should be tempered in light of more recent studies [2]. The literature nonetheless agrees that obesity represents a clinical challenge requiring high-risk management, particularly given the increased complications associated with reintubation [34]. Prophylactic NIV therefore appears to be a valuable strategy to improve outcomes in this population.

Steier et al. [35] previously reported in healthy volunteers’ subjects with obesity that the application of CPAP reduced diaphragm electromyogram and inspiratory pressure swings by 40% and 25%, respectively. Furthermore, when applying PSV-PEEP, we showed a decrease in inspiratory WOB when compared to spontaneous breathing or CPAP. These results may partly be explained by a reduction in intrinsic PEEP when applying positive airway pressure. In morbidly obese patients, tidal expiratory flow limitation is common [36, 37] and can promote dynamic hyperinflation and increased WOB. Upper airway inflammation immediately after extubation [38] may further contribute, particularly when combined with obesity-related anatomical changes, favoring airway collapse or worsening of unrecognized sleep apnea. However, in our study the difference in PEEPi between standard oxygen and CPAP or PSV-PEEP was limited (1–2 cmH₂O) and is unlikely to be clinically significant.

To our knowledge, the higher potential benefit of PSV-PEEP versus CPAP in recently extubated patients with morbid obesity (Figs. 2, 3) were poorly explored in physiological studies. One study showed that higher PEEP levels are needed in patients with morbid obesity compared to patients without obesity, during acute hypercapnic respiratory failure [39]. Beyond obesity, our physiological findings are consistent with prior [40] and recent [41] studies demonstrating the impact of PSV-PEEP in reducing inspiratory effort after extubation. Studies in acute hypoxemic respiratory failure, such as that of Menga et al. [42], including the comparison with CPAP, further support the role of pressure support in unloading respiratory muscles. Our results confirm that the addition of pressure support to PEEP provides a greater reduction in effort than CPAP alone, underlining the specific contribution of PSV in this setting.

Our study presents several limitations. This physiological study assessed the indexes of inspiratory effort twenty minutes after extubation for a twenty-minute period and the study was not designed to explore long-term consequences of NIV on outcome or extubation failure. The physiological results based on respiratory mechanics simply observed a decrease of each parameter (WOB, PTP, esophageal swings) when applying a positive airway pressure. This study did not investigate the duration of NIV need nor the outcome of patient receiving NIV. Although, recent studies suggest a benefit in NIV use when applying a high regimen of 6–12 h during the first 48H after extubation [16–18]. Second, the absence of random ordering and washout period could bias the results, as the prior condition could have influenced respiratory effort during the subsequent one by partially unloading the patient. To mitigate this potential effect, only the last 10 interpretable respiratory cycles of each phase were retained for analysis. Third, the levels of PSV and PEEP were not identical across all patients. As protocolized in our unit, ventilatory settings were maintained as prescribed prior to extubation. However, the mean PEEP level was 7 ± 2 cmH₂O, which is comparable to the PEEP used in CPAP mode. This allows us to specifically evaluate the effect of adding pressure support over the same level of PEEP provided in CPAP modality. Fourth, we acknowledge that the use of a facial mask for measurements may have influenced respiratory effort. Nevertheless, as the same mask was applied consistently across all three modalities (oxygen, CPAP, and PSV-PEEP), the validity of the comparisons was preserved. Fifth, the use of HFNO following extubation was not assessed. Recent physiological evidence suggests that HFNO may offer a benefit by reducing the work of breathing compared to conventional oxygen therapy following extubation [43]. However, NIV seems more effective [41], and according to recent clinical data [16–18], the use of HFNO to prevent reintubation in patients with obesity remains controversial. There is ongoing debate and uncertainty about the effectiveness of using HFNO to prevent reintubation in critically ill patients with obesity. This highlights the need for further research and consideration of the available evidence to make informed decisions about the use of HFNO in this specific patient population.

## Conclusion

The present study reports for the first time physiological evaluation of respiratory mechanic parameters for critically ill patients with morbid obesity, during post-extubation period. Our results suggest a significant decrease in all inspiratory effort indexes and especially in inspiratory WOB when applying NIV with CPAP and even more with PSV-PEEP. These results emphasize the pivotal role of pressure support, in addition to PEEP, in the management of morbidly obese patients after extubation, and may help guide the prophylactic use of NIV in this high-risk population.

## Supplementary Information

**Additional file 1.**

## Data Availability

The datasets used and/or analysed during the current study are available from the corresponding author on reasonable request.

## Abbreviations

BMI: Body Mass Index
CPAP: Continuous Positive Airway Pressure
FiO2: Inspired Oxygen Fraction
ICU: Intensive Care Unit
HFNO: High Flow Nasal Oxygen
MV: Minute Ventilation
NIV: Noninvasive Ventilation
Pao: Pressure at airway opening
Pdi: Transdiaphragmatic Pressure
PEEP: Positive End Expiratory Pressure
PEEPi: Intrinsic Positive End Expiratory Pressure
Pes: Esophageal pressure
Pga: Gastric pressure
PSV: Pressure Support Ventilation
PTPes: Esophageal Pressure Time Product
PTPdi: Diaphragmatic Pressure Time Product
RR: Respiratory Rate
SAPS II: Simplified Acute Physiology Score II
Td: Trigger delay
Te: Expiratory time
Ti: Inspiratory Time
Ttot: Total Cycle Duration
VE: Volume per minute
VT: Tidal Volume
VTe: Expired Tidal Volume
WOB: Work of Breathing
