# Supplementary material for: Physiological comparison of noninvasive ventilation and CPAP on inspiratory efforts after extubation in critically ill patients with morbid obesity: a post-hoc analysis
Source: Ann Intensive Care. 2025 Nov 17;15:180. doi: 10.1186/s13613-025-01603-3 (PMC12623563; doi:10.1186/s13613-025-01603-3)

# SUPPLEMENTARY MATERIAL

**Physiological comparison of noninvasive ventilation and CPAP on inspiratory efforts after extubation in critically ill patients with morbid obesity**

Martin Mahul, Mathieu Capdevila, Fabrice Galia, Audrey De Jong, Samir Jaber

**Table of contents**

- **Table S1:** Individual characteristics of the patients
- **Table S2:** Friedman test results for indices of inspiratory effort.
- **Table S3**: Pairwise Wilcoxon signed-rank tests with Holm correction for inspiratory effort indices.
- **Figure S1**: Flow chart of the study
- **Figure S2**: Individual and mean changes in Respiratory rate
- **Figure S3**: Individual and mean changes in Respiratory rate over tidal volume (RR/VT).

**Table S1.** Individual characteristics of the patients

| **Patient** | **Sex** | **Age** | **SAPSII** | **Height**  **(cm)** | **Weight**  **(kg)** | **BMI**  **(kg/m^2^)** | **Underlying**  **diseases** | **Etiology of respiratory failure** | **ETT**  **(mm)** | **MV before extubation**  **(days)** | **PSV level before extubation**  **(cmH2O)** | **PEEP before extubation**  **(cmH2O)** | **Extubation Failure** | **Outcome (D/S)** |
| --- | --- | --- | --- | --- | --- | --- | --- | --- | --- | --- | --- | --- | --- | --- |
| 1 | F | 85 | 68 | 163 | 115 | 43 | NIDDM | Pneumonia | 7.5 | 4 | 15 | 7 | N | S |
| 2 | M | 64 | 50 | 170 | 130 | 44 | NIDDM | Pancreatitis | 8 | 3 | 12 | 8 | N | S |
| 3 | F | 59 | 60 | 155 | 95 | 39 | None | Peritonitis | 7.5 | 3 | 12 | 8 | N | S |
| 4 | F | 49 | 66 | 160 | 174 | 67 | COPD, OSA | Sepsis | 7.5 | 6 | 10 | 10 | N | S |
| 5 | F | 25 | 29 | 172 | 145 | 49 | None | Asthma | 7.5 | 1 | 10 | 8 | N | S |
| 6 | F | 54 | 19 | 153 | 121 | 51 | Asthma, HTN | Postoperative | 7.5 | 1 | 10 | 8 | N | S |
| 7 | M | 37 | 54 | 180 | 130 | 40 | None | Pancreatitis | 8 | 14 | 8 | 10 | N | S |
| 8 | F | 78 | 90 | 155 | 87 | 36 | None | Bowel obstruction | 7.5 | 4 | 8 | 5 | N | S |
| 9 | F | 49 | 78 | 167 | 112 | 41 | Asthma, OSA | Peritonitis | 7.5 | 30 | 8 | 5 | N | S |
| 10 | F | 73 | 77 | 150 | 93 | 41 | CHF, AF | Septic shock | 7.5 | 4 | 12 | 6 | N | D |
| 11 | F | 50 | 45 | 162 | 94 | 36 | None | Necrotizing Fasciitis | 7.5 | 2 | 9 | 7 | N | S |
| 12 | M | 63 | 64 | 175 | 180 | 56 | NIDDM, HTN | Small bowel bleeding | 7.5 | 1 | 8 | 10 | N | S |
| 13 | F | 43 | 48 | 155 | 105 | 43 | OSA | Pneumonia | 7.5 | 3 | 12 | 7 | N | S |
| 14 | F | 77 | 41 | 155 | 84 | 36 | NIDDM, HTN | Pancreatitis | 7.5 | 7 | 9 | 7 | Y | S |
| 15 | F | 50 | 64 | 164 | 124 | 46 | OSA | Postoperative | 7.5 | 8 | 14 | 8 | N | S |
| Overall | 80%  F | 54  [49-68] | 60  [46-67] | 162  [155-168] | 115  [94-130] | 43  [39-47] | . | . | 87%  7.5 | 4  [2-6] | 10  [8-12] | 8  [7-8] | 93%  N | 93%  S |

Continuous variables are presented as median [interquartile range] and categorical variables as number (percentage).

Definition of abbreviations: AF: Atrial Fibrillation, BMI: Body Mass Index; CHF: Chronic Heart Failure, COPD: Chronic Obstructive Pulmonary Disease, D: Deceased; ETT: Endotracheal Tube Internal Diameter; F: female; M: male; HTN: Hypertension; MV: Mechanical Ventilation; NIDDM: Non Insulin Dependent Diabetes Mellitus; OSA: Obstructive Sleep Apnoea, PEEP: Positive End Expiratory Pressure; PSV: Pressure Support Ventilation; SAPS II Simplified Acute Physiology Score II ([2](#_ENREF_2)); S: Survived. All postoperative cases occurred after abdominal surgery, and all episodes of pancreatitis were acute.

**Table S2:** Friedman test results for indices of inspiratory effort.

| **Inspiratory effort indice** | **Friedman test: χ²(2)** | **p-value** |
| --- | --- | --- |
| WOB (J/min) | 28.1 | 7.8 × 10⁻⁷ |
| WOB (J/L) | 30.0 | 3.1 × 10⁻⁷ |
| Swing Pdi | 28.1 | 7.8 × 10⁻⁷ |
| Swing Pes | 29.5 | 3.9 × 10⁻⁷ |
| PTPdi | 30.0 | 3.1 × 10⁻⁷ |
| PTPes | 30.0 | 3.1 × 10⁻⁷ |

**Table S3.** Pairwise Wilcoxon signed-rank tests with Holm correction for inspiratory effort indices.

| **Inspiratory effort indice** | **Comparison** | **Raw p-value** | **Holm-adjusted p-value** | **Significant** |
| --- | --- | --- | --- | --- |
| WOB (J/min) | O₂ vs CPAP | 0.00012 | 0.00018 | Yes |
|  | O₂ vs PSV-PEEP | 0.00006 | 0.00018 | Yes |
|  | CPAP vs PSV-PEEP | 0.00006 | 0.00018 | Yes |
| WOB (J/L) | O₂ vs CPAP | 0.00006 | 0.00018 | Yes |
|  | O₂ vs PSV-PEEP | 0.00006 | 0.00018 | Yes |
|  | CPAP vs PSV-PEEP | 0.00006 | 0.00018 | Yes |
| Swing Pdi | O₂ vs CPAP | 0.00006 | 0.00018 | Yes |
|  | O₂ vs PSV-PEEP | 0.00006 | 0.00018 | Yes |
|  | CPAP vs PSV-PEEP | 0.00012 | 0.00018 | Yes |
| Swing Pes | O₂ vs CPAP | 0.00006 | 0.00018 | Yes |
|  | O₂ vs PSV-PEEP | 0.00006 | 0.00018 | Yes |
|  | CPAP vs PSV-PEEP | 0.00097 | 0.00097 | Yes |
| PTPdi | O₂ vs CPAP | 0.00006 | 0.00018 | Yes |
|  | O₂ vs PSV-PEEP | 0.00006 | 0.00018 | Yes |
|  | CPAP vs PSV-PEEP | 0.00006 | 0.00018 | Yes |
| PTPes | O₂ vs CPAP | 0.00006 | 0.00018 | Yes |
|  | O₂ vs PSV-PEEP | 0.00006 | 0.00018 | Yes |
|  | CPAP vs PSV-PEEP | 0.00006 | 0.00018 | Yes |

**Figure S1**: Flow chart of the study


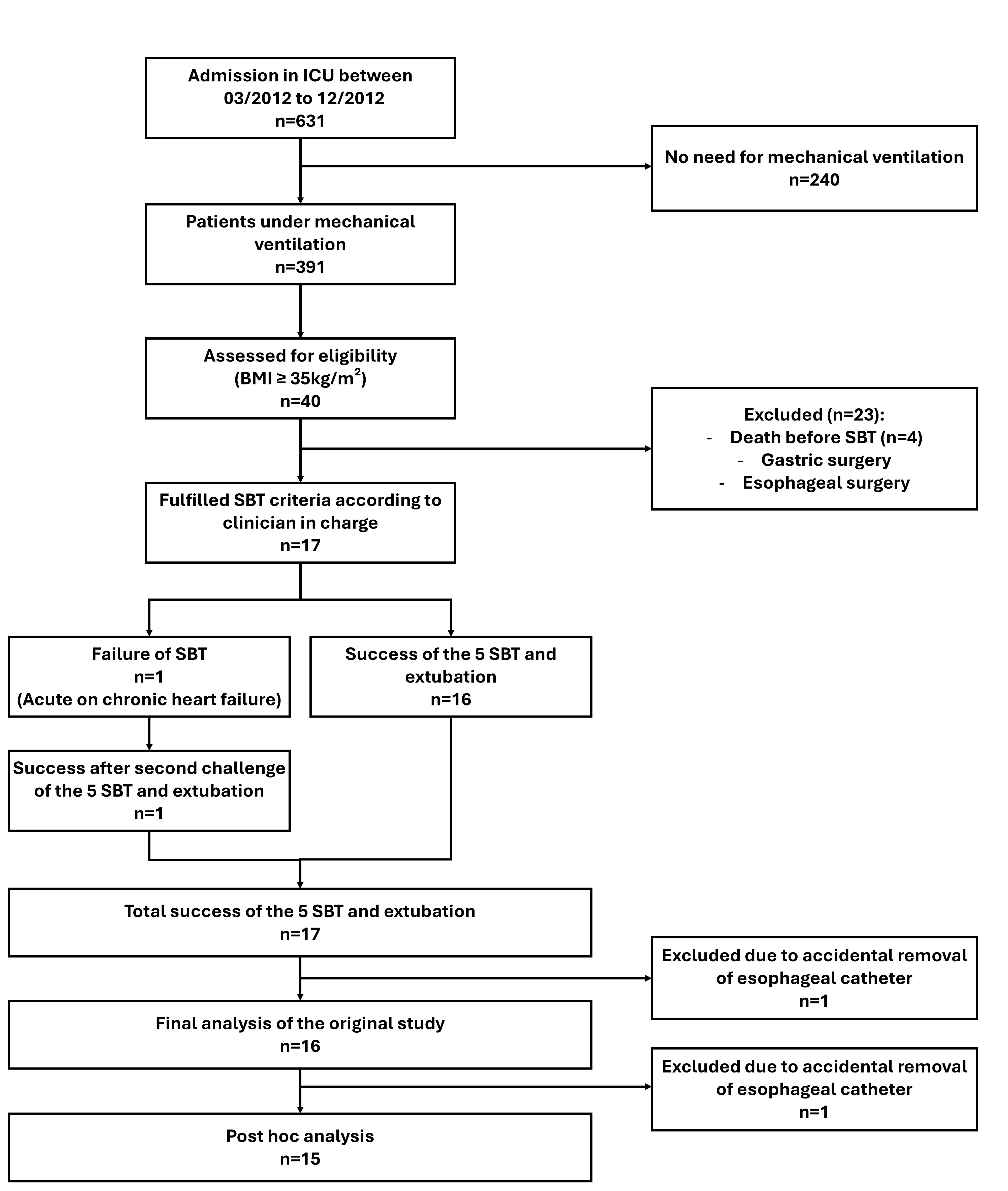


**Figure S2:** Individual and mean changes in Respiratory rate. * p< 0.05


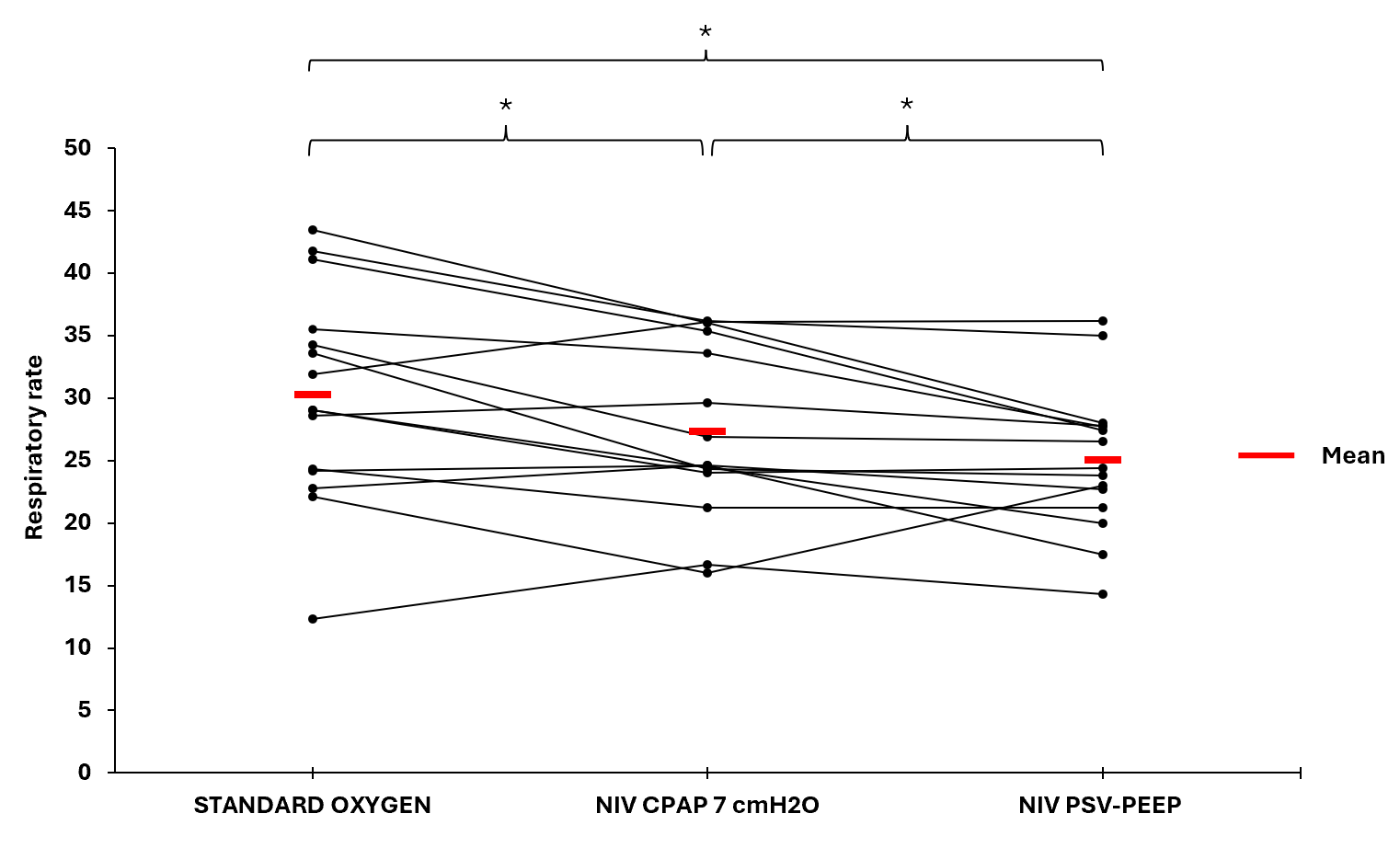


**Figure S3:** Individual and mean changes in Respiratory rate over tidal volume (RR/VT). * p< 0.05


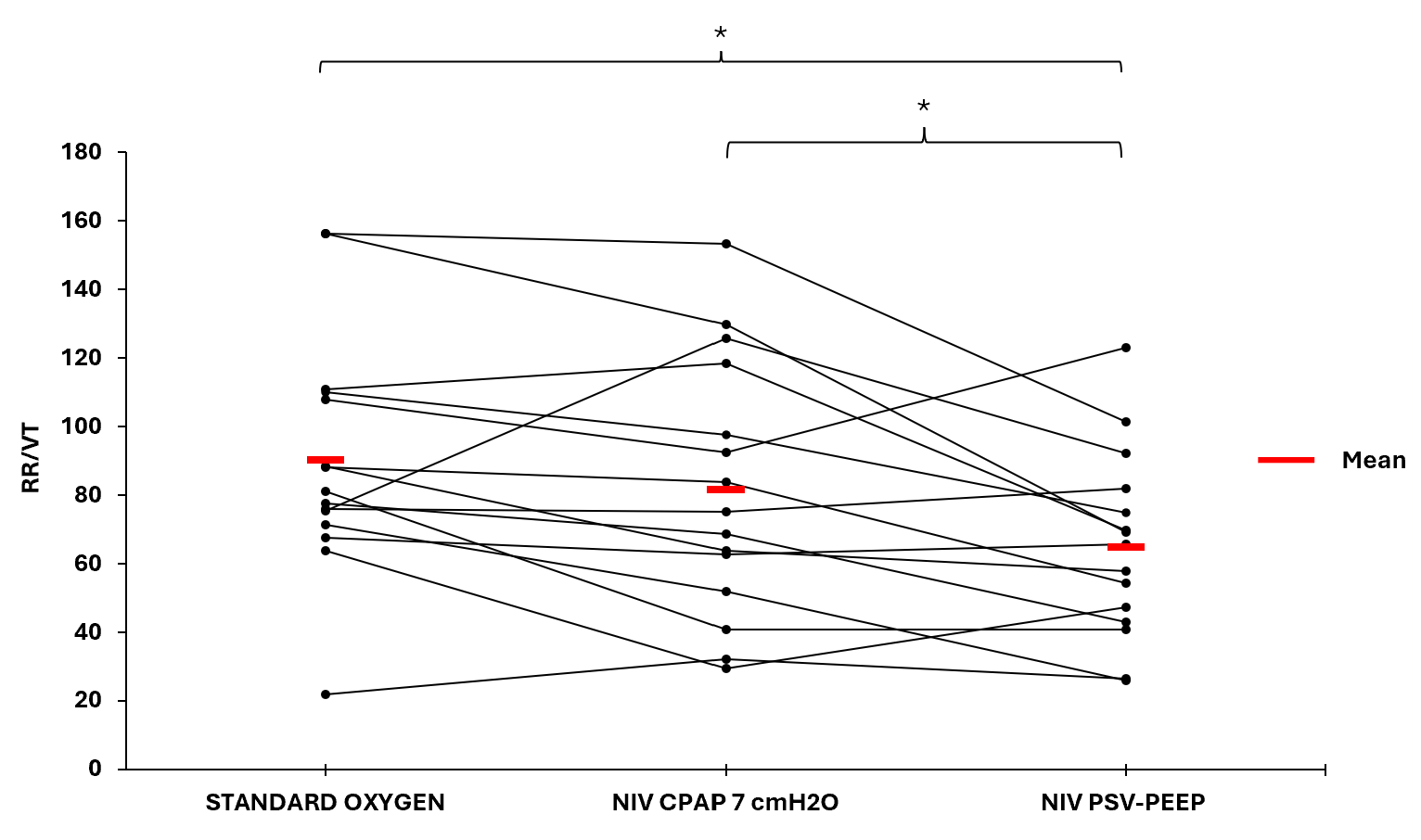

Supplement: Supplementary file 1 — Additional file 1. [file 13613_2025_1603_MOESM1_ESM.docx]
